# The relationship between self-regulated learning, mindful agency, and psychological resilience in Chinese master of nursing specialists: A cross-sectional study

**DOI:** 10.3389/fpsyg.2023.1066806

**Published:** 2023-03-14

**Authors:** Rui Yang, YuFang Gao, ZiTong Ji

**Affiliations:** ^1^The Affiliated Hospital of Qingdao University, Qingdao, China; ^2^School of Nursing, Qingdao University, Qingdao, China

**Keywords:** self-regulated learning, mindful agency, psychological resilience, master of nursing specialists, cross-sectional study

## Abstract

**Background:**

Self-regulated learning helps to improve academic performance and is an important strategy for the sustainable development of Master of Nursing Specialists. Consequently, it is relevant to identify the factors that affect self-regulated learning and analyze the correlation between them.

**Objective:**

This study examined the status quo of self-regulated learning, the relationship between self-regulated learning, mindful agency, and psychological resilience, and explored whether mindful agency and psychological resilience influence self-regulation learning.

**Methods:**

Chinese Master of Nursing Specialists were recruited to participate in an online survey from March to November 2022. Self-regulated learning, mindful agency, and psychological resilience were measured using three questionnaires, including the Self-Regulated Learning Scale for Clinical Nursing Practice Scale (SRLS-CNP), the Mindful Agency Scale, and the 10-item Connor-Davidson Resilience Scale (CD-RISC-10). The data were processed and analyzed using SPSS26.0. The statistical methods included descriptive statistics, Pearson’s correlation analyses, and multiple linear regression.

**Results:**

Self-regulated learning of Chinese Master of Nursing Specialists was medium level (59.24 ± 9.33 scores). Mindful agency and psychological resilience were positively correlated with self-regulated learning (*p* < 0.01) and important predictors of self-regulated learning of Master of Nursing Specialists, explaining 44.6% of the variation.

**Conclusion:**

Mindful agency and psychological resilience affected the self-regulated learning level of Master of Nursing Specialists in clinical practice. These results will enable clinical educators to pay more attention to the personal psychological factors of Master of Nursing Specialists to improve their self-regulated learning ability through mindful agency and psychological resilience.

## 1. Introduction

Lifelong learning is a pillar of nursing practice and a conscious pursuit of caregivers throughout their careers, helping caregivers prepare for new or different roles and respond positively to the inevitable changes in clinical practice (Bindon, 2017), such as the continuous rapid development of medical technology, the increasing demand for services related to the aging population (Ge et al., 2020) and the spread of infectious and chronic diseases (Baker et al., 2021). Self-regulated learning (SRL) refers to regulating emotion, cognition, and behavior throughout the learning process to achieve at the expected level and is the main feature of lifelong learning (Casali et al., 2022; Albani et al., 2023). Self-regulating learning skills are particularly important in complex clinical learning environments. Self-regulation has a beneficial effect of improving medical students’ academic performance (Wang et al., 2020; Ballouk et al., 2022) and clinical skills (Goldowsky and Rencic, 2022), improving their mental health (Baars et al., 2017), and is an important strategy for the lifelong career development of medical students.

In 2010, the State Council of China approved the establishment of the professional degree of Master of Nursing Specialist (MNS) to cultivate high-level, application-oriented professionals with strong professional practical ability and theoretical knowledge who can creatively engage in specialized nursing practice (ADCSC, 2010). Studies have demonstrated that MNS graduates have gradually become leaders of clinical nursing reform and coordinators of the multidisciplinary health team (Goldsberry, 2018). Training nursing students’ self-regulated learning ability in clinical nursing practice significantly improves their autonomy, self-confidence, and lifelong learning ability (Iyama and Maeda, 2018). In this regard, identifying the self-regulated learning of MNS graduates and the factors that affect it will be useful during their clinical practice in the clinical learning environment.

Studies have depicted that learners’ skills in regulating mechanisms (such as attention, emotional control, planning, metacognition, etc.) are closely related to self-regulating learning (van der Riet et al., 2018). Mindful agency refers to a positive mental quality where learners regulate their emotional ability and their cognitive process of learning through mindfulness (Wang and Peng, 2017). Nursing students with higher mindful agency have a better psychological status helping them to better adapt to the clinic (Kakoschke et al., 2021). Mindfulness intervention can reduce anxiety and stress, thereby increasing trainee nursing students’ happiness (Dai et al., 2022). It has been reported that the mindful agency of nursing students positively impacts their regulatory behavior since mindful agency can promote cognition (Fabio and Towey, 2018), emotional regulation (Lee and Jang, 2021), self-awareness, and improve attention regulation (Wang and Peng, 2017).

Psychological resilience is the ability of individual learners to overcome difficulties, mobilize learning motivation and deal with external pressure factors in the face of adversity (Sisto et al., 2019), which can guide students to face clinical challenges, improve nursing quality, their psychological status and health (Cleary et al., 2018; Ríos-Risquez et al., 2018). Indeed, nursing students with higher psychological resilience tend to adopt positive ways to cope with stress and demonstrate better clinical adaptability (Amsrud et al., 2019; Li and Hasson, 2020). Mao et al. (2022) found that psychological resilience plays a positive role in arousing positive emotions. In addition, Liu et al.’s (2021) research found that psychological resilience is a protective factor and positively correlates with emotional regulation ability.

Recently, some studies have explored the self-regulated learning of medical students and the factors influencing self-regulated learning (van Houten-Schat et al., 2018). However, there is a lack of research on the relationship between mindful agency, psychological resilience, and the clinical self-regulation learning level of Master of Nursing Specialists in the context of China’s higher nursing education. Therefore, this study aimed to explore the relationship between mindful agency, psychological resilience, and clinical self-regulated learning level of Master of Nursing Specialists. Understanding the influencing factors of self-regulated learning can better cultivate Master of Nursing Specialists. It is hypothesized that mindful agency and psychological resilience are related to self-regulated learning of the clinical nursing practice of Master of Nursing Specialists during the clinical period.

## 2. Methods

### 2.1. Participants

From March 2022 to November 2022, Master of Nursing Specialists from Shandong, Liaoning, and other higher nursing institutions were recruited. The inclusion criteria were: (a) full-time postgraduate nursing student; (b) participation in clinical practice; (c) willingness to participate in the study and provide informed consent. The exclusion criterion was those who did not complete the questionnaire.

The sample size was calculated according to the multivariate linear regression equation, and the sample size could be at least 10 to 15 times the number of variables in the equation (Trigo et al., 2018). There were 6 independent variables that were introduced into the equation. Thus, the sample size was at least 60–90, and considering the unavailability and a nonresponse rate of 20%, the required amount for the sample was 75–113. In the present study, there were 216 participants in total. All participants were invited to complete the survey through network links, and 196 valid questionnaires were collected.

### 2.2. Procedure

The researchers sent QR codes through China’s social media networks, and the participants completed the survey through “WJX”.^1^ The questionnaire consisted of three parts: informed consent, general information, and self-reporting scales.

### 2.3. Ethical considerations

This study was approved by the ethics committee of QingDao University (QDU-HEC-2021102), and all participation was voluntary and anonymous. This study was conducted in accordance with the Declaration of Helsinki.

### 2.4. Instruments

#### 2.4.1. The self-regulated learning scale for clinical nursing practice scale (SRLS-CNP)

The Master of Nursing Specialists’ self-regulated learning was assessed by the SRLS-CNP that was developed by Iyama and Maeda (2018) (Cronbach’s α = 0.85) and translated into Chinese by Chen et al. (2021). The scale has good reliability and validity, with Cronbach’s α of 0.940. The scale comprises sixteen items and two factors, with each item fully reflecting the concept connotation of self-regulated learning in nursing students’ clinical practice. Each item is rated on a 5-point Likert-type scale ranging from 1 ‘strongly disagree’ to 5 ‘strongly agree’, and the composite scores range from 16 to 80. We took a score of 48 as the cut-off value and divided the MNS into lower self-regulated learning level group (<48 scores), medium self-regulated learning level group (48–64 scores), and higher self-regulated learning level group (>64 scores). The higher the score, the stronger the self-regulated learning ability of nursing students. In this study, Cronbach’s α was 0.938.

#### 2.4.2. The mindful agency scale

Mindful agency was measured using the Chinese mindful agency scale based on the mindful agency subscale used by Deakin et al. (2015) in analyzing the learning power model (Wang and Peng, 2017). The tool has five dimensions, including sixteen items: learning methods (3 items), emotional regulation (2 items), awareness of planning (3 items), openness to experience (5 items), and learning engagement (3 items). The scale has good reliability and validity, with Cronbach’s α of 0.840. The mindful agency scale is scored using a 6-point Likert-type scale ranging from 1 ‘strongly inconsistent’ to 6 ‘strongly consistent’. The total scale ranges from 16 to 96, with higher scores indicating a higher level of mindful agency. We took a score of 57.6 as the cut-off value and divided the MNS into lower mindful agency group (<57.6 scores), medium mindful agency group (57.6–76.8 scores), and higher mindful agency group (>76.8 scores). In this study, Cronbach’s α was 0.957.

#### 2.4.3. 10-item Connor-Davidson resilience scale (CD-RISC-10)

Psychological resilience was evaluated using the CD-RISC-10 developed by Campbell-Sills and Stein (2007), which was revised and translated into Chinese by Ye et al. (2016). The Chinese CD-RISC-10 consists of one dimension, including ten items and has sound reliability and validity (Cronbach’s α = 0.851). The CD-RISC-10 scale is scored using a 5-point Likert-type scale ranging from 0 to 4 with a total score ranging from 0 to 50. We took a score of 30 as the cut-off value and divided the MNS into lower psychological resilience group (<30 scores), medium psychological resilience group (30–40 scores), and higher psychological resilience group (>40 scores). The higher scores indicate a higher level of psychological resilience. In this study, Cronbach’s α was 0.934.

The Master of Nursing Specialists’ demographic characteristics, such as age, sex, grade, clinical practice time, graduation employment intention, etc., were collected by the General Information Questionnaire.

### 2.5. Statistical analysis

The data were analyzed using the statistical SPSS 26.0 software (IBM, Armonk, NY, United States). Descriptive statistics were used to analyze the demographic characteristics. The data were tested for skewness and kurtosis and the scores of self-regulated learning, mindful agency and psychological resilience conform to a normal distribution, so they are described by means and standard deviation. The relationships among the main variables and their components were examined by Pearson’s correlation analyses. Multiple linear regression was used to analyze the influencing factors of self-regulated learning (*α* = 0.05).

## 3. Results

### 3.1. Demographic characteristics

Of the 216 Master of Nursing Specialists recruited, 196 completed the questionnaire (90.74% response rate). There were 76 Grade 1 Master of Nursing Specialists, 85 Grade 2 Master of Nursing Specialists, and 35 Grade 3 Master of Nursing Specialists. Their demographic characteristics are provided in Table 1.

**Table 1:** Participants’ demographic characteristics (*N* = 196).

Characteristics	*N*	%	*p*
Age			0.757
20~	156	79.59	
26~	40	20.41	
Sex			0.691
Male	26	13.27	
Female	170	86.73	
Year of study			0.452
Number of Grade 1	76	38.78	
Number of Grade 2	85	43.37	
Number of Grade 3	35	17.85	
Clinical practice time			0.177
<6 months	43	21.94	
6–12 months	51	20.02	
>12 months	102	52.04	

### 3.2. Self-regulated learning, mindful agency, and psychological resilience

The mean scores, standard deviations, and correlations among the study variables and their components are presented in Table 2, showing that mindful agency and psychological resilience positively correlated with self-regulated learning (*p* < 0.05).

**Table 2:** Means, standard deviations, and correlations among the study variables (*N* = 196).

Variables	Mean ± SD	1	2	3	4	5	6	7	8	9	10
1. Motivation	3.56 ± 0.68	1	–	–	–	–	–	–	–	–	–
2. Learning strategy	3.81 ± 0.58	0.77^**^	1	–	–	–	–	–	–	–	–
3. Self-regulated learning	3.70 ± 0.58	0.94^**^	0.95^**^	1	–	–	–	–	–	–	–
4. Learning method	4.57 ± 0.78	0.52^**^	0.68^**^	0.63^**^	1	–	–	–	–	–	–
5. Emotion regulation	4.32 ± 0.92	0.40^**^	0.46^**^	0.46^**^	0.77^**^	1	–	–	–	–	–
6. Planning consciousness	4.57 ± 0.76	0.47^**^	0.63^**^	0.59^**^	0.71^**^	0.70^**^	1	–	–	–	–
7. Openness to experience	4.46 ± 0.73	0.51^**^	0.62^**^	0.61^**^	0.66^**^	0.64^**^	0.74^**^	1	–	–	–
8. Learning Engagement	4.39 ± 0.77	0.28^**^	0.44^**^	0.39^**^	0.57^**^	0.45^**^	0.61^**^	0.65^**^	1	–	–
9. Mindful agency	4.47 ± 0.66	0.52^**^	0.67^**^	0.64^**^	0.86^**^	0.81^**^	0.88^**^	0.90^**^	0.78^**^	1	–
10. Psychological resilience	3.67 ± 0.61	0.54^**^	0.61^**^	0.62^**^	0.62^**^	0.55^**^	0.68^**^	0.70^**^	0.60^**^	0.75^**^	1

^**^At the 0.01 level, the correlation was significant.

### 3.3. Mindful agency and psychological resilience influence self-regulation learning

The multiple linear regression analysis results are provided in Table 3, showing that mindful agency and psychological resilience can predict 44.6% of the level of self-regulated learning.

**Table 3:** Simultaneous multiple regression model results (2 independent variables enter model together).

Independent variable	Standardized coefficients	Standard errors	*t* statistic	*p*-Value
(constant)	16.330	3.461	4.719	0.001
Mindful agency	0.359	0.071	5.056	0.001
Psychological resilience	0.471	0.122	3.852	0.001

Adjusted *R*^2^ = 0.446; F-Statistic for overall model = 79.445; *p* < 0.001.constant:self-regulated learning.

## 4. Discussion

This study explored the status quo of clinical self-regulated learning of Chinese Master of Nursing Specialists, as well as the impact of mindful agency and psychological resilience on the level of clinical self-regulated learning.

The clinical self-regulation learning level of nursing graduate students in this study was medium level, and the motivation dimension score was relatively low. These findings are consistent with Mäenpää et al.’s (2020) and Chen et al.’s (2019) studies. The relatively low sense of self-efficacy of some nursing graduate students may be the main reason why clinical self-regulated learning is at the medium level, and the score of motivation dimension is low (Fernandez-Rio et al., 2017). After some students entered clinical study, their role orientation was still unclear (Cretu and Stilos, 2021). In addition, in the context of the normalization of the COVID-19 pandemic, the complex clinical learning environment, how to balance the pressure of hospital and school life (Laurencelle and Scanlan, 2018), and heavy workload may lead some graduate students to self-doubt, reducing their confidence and enthusiasm for education, so they lack the motivation to complete their studies.

Mindful agency is positively correlated with the clinical self-regulated learning level of Master of Nursing Specialists, which is consistent with previous research results. For example, motivation and attention are positive personal influencing factors affecting self-regulated learning since students with higher intrinsic motivation and concentration had a better level of clinical self-regulated learning (Berkhout et al., 2015; Baars et al., 2017; van der Riet et al., 2018). Armstrong (2022) reported that the level of students’ mindfulness affects their attention, with mindful learners effectively filtering interfering information and improving learning engagement. Meanwhile, the study by Donald et al. (2020) found a positive relationship between mindfulness and intrinsic motivation. Cultivating the mindfulness subjectivity of Master of Nursing Specialists can improve their learning attention, promote their learning interest, stimulate their internal learning motivation, and improve their self-regulated learning in clinical practice. Mindful agency positively impacts focusing attention, improving mental health, and improving patient care. Therefore, many researchers have suggested further improving students’ mindful agency (van Houten-Schat et al., 2018). Mindfulness training (such as body scanning, mindfulness meditation, mindfulness exercise, and stress reduction training) can be effectively brought into formal practice, and more importantly, integrated into informal practice. At the same time, carry out peer assistance programs to share experiences with peers.

Psychological resilience is positively correlated with clinical self-regulated learning of Master of Nursing Specialists. The study found that low psychological resilience may increase the psychological distress of nursing students, so they cannot effectively deal with external pressure in the face of adversity. Furthermore, previous studies have demonstrated that the psychological resilience of nursing students is related to their problem-solving ability (Pinar et al., 2018), adaptability, and mental health (Li and Hasson, 2020), which may further affect their self-regulated learning in clinical practice. The nursing students with psychological resilience showed stronger perseverance and an optimistic attitude and could actively face the difficulties encountered in the clinical environment to improve their clinical adaptability and enhance their sense of happiness (Meyer et al., 2020). The present study demonstrated that psychological resilience needs to be cultivated in clinical nursing education to better improve nurses’ clinical adaptability and self-regulation ability, thereby improving their sense of happiness and promoting patient safety.

## 5. Limitations

This study has some limitations. First, it is a cross-sectional study, and the results may be biased due to data collected *via* electronic questionnaires. Future studies should investigate the factors influencing self-regulated learning in the form of interviews. Second, the number of samples included in this study was small, so it is necessary to further expand the sample size in the future.

## 6. Conclusion

The clinical practice self-regulation ability of Master of Nursing Specialists was found to be at a medium level and positively related to the level of mindful agency and psychological resilience. In addition, it was found that the level of mindful agency and psychological resilience of the Master of Nursing Specialists influences the level of clinical self-regulated learning. These results will enable clinical educators to pay more attention to the personal psychological factors of Master of Nursing Specialists to improve their self-regulated learning ability through mindful agency and psychological resilience.

## Data availability statement

The original contributions presented in the study are included in the article/supplementary material, further inquiries can be directed to the corresponding author.

## Ethics statement

Written informed consent was obtained from the individual(s) for the publication of any potentially identifiable images or data included in this article.

## Author contributions

RY: data curation and writing–original draft. RY and ZJ: investigation. YG: supervision. All authors contributed to the article and approved the submitted version.

## Conflict of interest

The authors declare that the research was conducted in the absence of any commercial or financial relationships that could be construed as a potential conflict of interest.

## Publisher’s note

All claims expressed in this article are solely those of the authors and do not necessarily represent those of their affiliated organizations, or those of the publisher, the editors and the reviewers. Any product that may be evaluated in this article, or claim that may be made by its manufacturer, is not guaranteed or endorsed by the publisher.

## Footnotes

^1^ www.wjx.cn

## References

[ref1] ADCSC. (2010). Notice on the issuance of 19 professional degree setting programs, including the master of finance. Available at: http://www.moe.gov.cn/srcsite/A22/moe_833/201005/t20100513_92739.html (Accessed September 6, 2022).

[ref2] Albani A. Ambrosini F. Mancini G. Passini S. Biolcati R. (2023). Trait emotional intelligence and self-regulated learning in university students during the covid-19 pandemic: the mediation role of intolerance of uncertainty and covid-19 perceived stress. Pers. Individ. Dif. 203:111999. doi: 10.1016/j.paid.2022.111999, PMID: 36415560 PMC9671799

[ref3] Amsrud K. Lyberg A. Severinsson E. (2019). Development of resilience in nursing students: a systematic qualitative review and thematic synthesis. Nurse Educ. Pract. 41:102621. doi: 10.1016/j.nepr.2019.102621, PMID: 31726329

[ref4] Armstrong T. (2022). Mindfulness in the Classroom: Strategies for Promoting Concentration, Compassion, and Calm: Beijing: Chinese Youth Press.

[ref5] Baars M. Wijnia L. Paas F. (2017). The association between motivation, affect, and self-regulated learning when solving problems. Front. Psychol. 8:1346. doi: 10.3389/fpsyg.2017.01346, PMID: 28848467 PMC5550677

[ref6] Baker C. Cary A. Da C. B. M. (2021). Global standards for professional nursing education: the time is now. J. Prof. Nurs. 37, 86–92. doi: 10.1016/j.profnurs.2020.10.001, PMID: 33674114 PMC7571445

[ref7] Ballouk R. Mansour V. Dalziel B. McDonald J. Hegazi I. (2022). Medical students' self-regulation of learning in a blended learning environment: a systematic scoping review. Med. Educ. Online 27:2029336. doi: 10.1080/10872981.2022.2029336, PMID: 35086439 PMC8803058

[ref8] Berkhout J. Helmich E. Teunissen P. (2015). Exploring the factors influencing clinical students' self-regulated learning. Med. Educ. 49, 589–600. doi: 10.1111/medu.12671, PMID: 25989407

[ref9] Bindon S. (2017). Professional development strategies to enhance nurses' knowledge and maintain safe practice. AORN J. 106, 99–110. doi: 10.1016/j.aorn.2017.06.002, PMID: 28755675

[ref10] Campbell-Sills L. Stein M. (2007). Psychometric analysis and refinement of the Connor-Davidson resilience scale (cd-risc): validation of a 10-item measure of resilience. J. Trauma. Stress. 20, 1019–1028. doi: 10.1002/jts.20271, PMID: 18157881

[ref11] Casali N. Ghisi M. Meneghetti C. (2022). The role of general and study-related intraindividual factors on academic learning outcomes under covid-19: a cross-sectional and longitudinal analysis. Educ. Sci. 12:101. doi: 10.3390/educsci12020101

[ref12] Chen J. Björkman A. Zou J. Engström M. (2019). Self-regulated learning ability, metacognitive ability, and general self-efficacy in a sample of nursing students: a cross-sectional and correlational study. Nurse Educ. Pract. 37, 15–21. doi: 10.1016/j.nepr.2019.04.014, PMID: 31035075

[ref13] Chen Q. Wang J. Qian Z. Jin G. Zhang X. (2021). Reliability and validity of the chinese version of the self-regulated learning scale in clinical nursing practice. Chin. J. Modern Nurs. 27, 5021–5025. doi: 10.3760/cma.j.cn115682-20210610-02572

[ref14] Cleary M. Visentin D. West S. Lopez V. Kornhaber R. (2018). Promoting emotional intelligence and resilience in undergraduate nursing students: an integrative review. Nurse Educ. Today 68, 112–120. doi: 10.1016/j.nedt.2018.05.018, PMID: 29902740

[ref15] Cretu E. Stilos K. (2021). The value of a post-graduate clinical placement for nursing students. Can. Oncol. Nurs. J. 31, 494–499. 34786470 PMC8565439

[ref16] Dai Z. Jing S. Wang H. (2022). Mindfulness-based online intervention on mental health among undergraduate nursing students during coronavirus disease 2019 pandemic in Beijing, China: a randomized controlled trial. Front. Psych. 13:949477. doi: 10.3389/fpsyt.2022.949477, PMID: 36465283 PMC9709131

[ref17] Deakin C. Huang A. Goldspink S. (2015). Developing resilient agency in learning: the internal structure of learning power. Br. J. Educ. Stud. 63, 121–160. doi: 10.1080/00071005.2015.1006574

[ref18] Donald J. Bradshaw E. Ryan R. (2020). Mindfulness and its association with varied types of motivation: a systematic review and meta-analysis using self-determination theory. Personal. Soc. Psychol. Bull. 46, 1121–1138. doi: 10.1177/0146167219896136, PMID: 31884892

[ref19] Fabio R. Towey G. (2018). Long-term meditation: the relationship between cognitive processes, thinking styles and mindfulness. Cogn. Process. 19, 73–85. doi: 10.1007/s10339-017-0844-3, PMID: 29110263

[ref20] Fernandez-Rio J. Cecchini J. Méndez-Gimenez A. Mendez-Alonso D. Prieto J. (2017). Self-regulation, cooperative learning, and academic self-efficacy: interactions to prevent school failure. Front. Psychol. 8:22. doi: 10.3389/fpsyg.2017.00022 28154544 PMC5243853

[ref21] Ge Y. Wang L. Feng W. Zhang B. Liu S. Yh K. (2020). Challenges and strategies for healthy aging in China (in chinese). J Manag. World 36, 86–96. doi: 10.19744/j.cnki.11-1235/f.2020.0055

[ref22] Goldowsky A. Rencic J. (2022). Self-regulated learning and the future of diagnostic reasoning education. Diagnosis (Berl) 10, 24–30. doi: 10.1515/dx-2022-0066, PMID: 36476651

[ref23] Goldsberry J. (2018). Advanced practice nurses leading the way: interprofessional collaboration. Nurse Educ. Today 65, 1–3. doi: 10.1016/j.nedt.2018.02.024, PMID: 29518668

[ref24] Iyama S. Maeda H. (2018). Development of the self-regulated learning scale in clinical nursing practice for nursing students: consideration of its reliability and validity. Jpn. J. Nurs. Sci. 15, 226–236. doi: 10.1111/jjns.12191, PMID: 29152853

[ref25] Kakoschke N. Hassed C. Chambers R. Lee K. (2021). The importance of formal versus informal mindfulness practice for enhancing psychological wellbeing and study engagement in a medical student cohort with a 5-week mindfulness-based lifestyle program. PLoS One 16:e258999:e0258999. doi: 10.1371/journal.pone.0258999, PMID: 34673830 PMC8530308

[ref26] Laurencelle F. Scanlan J. (2018). Graduate students' experiences: developing self-efficacy. Int. J. Nurs. Educ. Scholarsh. 15, 2015–2018. doi: 10.1515/ijnes-2017-0041 29306923

[ref27] Lee M. Jang K. (2021). Nursing students' meditative and sociocognitive mindfulness, achievement emotions, and academic outcomes: mediating effects of emotions. Nurse Educ. 46, E39–E44. doi: 10.1097/NNE.0000000000000902, PMID: 32833880

[ref28] Li Z. Hasson F. (2020). Resilience, stress, and psychological well-being in nursing students: a systematic review. Nurse Educ. Today 90:104440. doi: 10.1016/j.nedt.2020.104440, PMID: 32353643

[ref29] Liu Y. Pan H. Yang R. (2021). The relationship between test anxiety and emotion regulation: the mediating effect of psychological resilience. Ann. General Psychiatry 20:40. doi: 10.1186/s12991-021-00360-4, PMID: 34488816 PMC8419945

[ref30] Mäenpää K. Järvenoja H. Peltonen J. Pyhältö K. (2020). Nursing students' motivation regulation strategies in blended learning: a qualitative study. Nurs. Health Sci. 22, 602–611. doi: 10.1111/nhs.12702, PMID: 32115833

[ref31] Mao C. Lin M. Shen S. Li Y. Xie Z. Li P. (2022). Latent profiles of emotion regulation strategies associated with alexithymia, nonsuicidal self-injury and resilience among nursing students. Stress. Health 38, 69–78. doi: 10.1002/smi.3075, PMID: 34152072

[ref32] Meyer G. Shatto B. Kuljeerung O. Nuccio L. Bergen A. Wilson C. (2020). Exploring the relationship between resilience and grit among nursing students: a correlational research study. Nurse Educ. Today 84:104246. doi: 10.1016/j.nedt.2019.104246, PMID: 31706204

[ref33] Pinar S. E. Yildirim G. Sayin N. (2018). Investigating the psychological resilience, self-confidence and problem-solving skills of midwife candidates. Nurse Educ. Today 64, 144–149. doi: 10.1016/j.nedt.2018.02.014, PMID: 29482050

[ref34] Ríos-Risquez M. García-Izquierdo M. Sabuco-Tebar E. Carrillo-Garcia C. Solano-Ruiz C. (2018). Connections between academic burnout, resilience, and psychological well-being in nursing students: a longitudinal study. J. Adv. Nurs. 74, 2777–2784. doi: 10.1111/jan.13794, PMID: 29992596

[ref35] Sisto A. Vicinanza F. Ampanozzi L. Ricci G. Tartaglini D. Tambone V. (2019). Towards a transversal definition of psychological resilience: a literature review. Medicina (Kaunas) 55:745. doi: 10.3390/medicina55110745, PMID: 31744109 PMC6915594

[ref36] Trigo T. de Freitas C. C. S. Wang Y. (2018). The influence of depression on the psychometric properties of the Maslach burnout inventory-human services survey: a cross-sectional study with nursing assistants. FPSYT 9:695. doi: 10.3389/fpsyt.2018.00695, PMID: 30618870 PMC6305309

[ref37] van der Riet P. Levett-Jones T. Aquino-Russell C. (2018). The effectiveness of mindfulness meditation for nurses and nursing students: an integrated literature review. Nurse Educ. Today 65, 201–211. doi: 10.1016/j.nedt.2018.03.018, PMID: 29602138

[ref38] van Houten-Schat M. Berkhout J. Van Dijk N. Endedijk M. Jaarsma A. Diemers A. (2018). Self-regulated learning in the clinical context: a systematic review. Med. Educ. 52, 1008–1015. doi: 10.1111/medu.13615, PMID: 29943415 PMC6175376

[ref39] Wang X. Li J. Wang C. (2020). The effectiveness of flipped classroom on learning outcomes of medical statistics in a Chinese medical school. Biochem. Mol. Biol. Educ. 48, 344–349. doi: 10.1002/bmb.21356, PMID: 32575169

[ref40] Wang Q. Peng Y. (2017). Mindful agency scale for college students. J. East China Normal Univ. 35, 146–154. doi: 10.16382/j.cnki.1000-5560.2017.05.013

[ref41] Ye Z. Ruan X. Zeng Z. Xie Q. Cheng M. Peng C.. (2016). Psychometric properties of 10-item connor-Davidson resilience scale among nursing stuidents. J. Nurs. 23, 9–13. doi: 10.3969/j.issn.1009-6493.2016.33.008

